# Lactational Performance of Early-Lactation Dairy Cows Fed Forages Produced by Two Different Crop Production Programs

**DOI:** 10.3390/ani15131836

**Published:** 2025-06-21

**Authors:** David P. Casper, Jon P. Pretz, Cliff Ramsier

**Affiliations:** 1 Casper’s Calf Ranch, 4890 West Lily Creek Road, Freeport, IL 61032, USA; 2 Department of Animal Sciences, North Carolina A&T State University, Greensboro, NC 27411, USA; 3 Hubbard Feeds, 111 West Cherry St. Suite 500, Mankato, MN 56001, USA; jonpretz@gmail.com; 4 Ag Spectrum, 428 E 11th St., DeWitt, IA 52742, USA; cliff@agspectrum.com

**Keywords:** forage digestibility, forage quality, high-forage ration, lactating dairy cows

## Abstract

Early-lactation high-producing dairy cows require highly digestible forages. Thirty early-lactation (58 days in milk; 38.9 kg/d milk) cows were blocked and randomly assigned to one of two treatments. The treatments were the control (**CON**), consisting of corn silage and alfalfa haylage produced via university crop production practices, versus supplementing soil and crop additives (**SCA**) during crop production. Milk production (32.6 and 36.9 kg/d for CON and SCA, respectively) increased (*p* < 0.04) when cows were fed SCA forages compared with cows fed CON forages, while the dry matter intakes were similar (*p* < 0.46). The yields of milk fat were similar (*p* > 0.26), but the milk protein (0.98 and 1.09 kg/d), lactose (1.62 and 1.88 kg/d), and total solids (3.77 and 4.25 kg/d) contents were greater (*p* < 0.05) for cows fed SCA compared with cows fed CON. The total-tract apparent neutral detergent fiber (48.5 and 54.7%) and acid detergent fiber (48.3 and 54.4%) digestibility increased (*p* < 0.03), while the starch digestibility (97.9 and 98.4%) tended (*p* < 0.06) to increase for cows fed SCA compared with cows fed CON. Feeding highly digestible forages during early lactation improved milk production, milk composition, and fiber digestibility in a high-forage ration.

## Simple Summary

A soil and crop additive crop production program (base saturation, biostimulates, and foliar-applied nutrients) to produce highly digestible forages could increase lactating dairy cow milk production when cows are fed a higher-forage ration. Thirty Holstein dairy cows at peak lactation (58 days in milk ± 2.9) were used to evaluate two agronomy crop production programs to grow corn silage and alfalfa haylage. The peak-lactation milk production, milk protein production, and fiber digestibility were greater for cows fed forage produced from the soil and crop additive agronomy crop production program. Crop production practices for producing forage can enhance milk production when cows are fed a higher-forage ration (61%) at peak lactation.

## 1. Introduction

In recent times, the scarcity or cost increases of commodities have placed more emphasis on forage nutrient digestibility. Oftentimes, these commodity scenarios increase the cost to produce 100 kg of milk, thereby exceeding market milk prices. Dairy cattle are biologically designed to convert forages and other fibrous feeds into high-quality milk and meat products [1,2]. Dairy producers are continuously forced to identify ways to reduce feed costs to maintain profitability [3,4]. The utilization of high-forage with lower-starch rations based on highly digestible forages can be one option to reduce feed costs, improve feed efficiency, and maintain profitability [2,5,6]. Forage nutrient digestibility is the basis for feeding higher-forage rations to meet the nutrient requirements of lactating dairy cows [7,8].

Forages can be the most economical nutrient source [3,9] in dairy operations, but there is a wide range of forage digestibility values within alfalfa and corn silage databases [3,9]. Casper and Mertens [7] demonstrated that the greatest factor affecting the conversion of feed to milk (feed efficiency) by a lactating dairy cow is nutrient digestibility, which directly impacts the ration energy density. Casper and Mertens [7] reported that nutrient digestibility is the greatest factor for meeting the nutrient requirements of high-producing dairy cows.

Dairy producers have improved their ability to consistently grow, harvest, and store highly digestible forages. Previous research [5,10,11,12] has focused on the use of high-forage rations for feeding lactating dairy cows to improve income over feed costs while potentially improving feed efficiency. Llameas0lamas and Combs [10] reported that highly digestible forages can be consumed in greater quantities (71.1 and 86% DM) compared with lower, more typical (55%) inclusion rates for feeding lactating dairy cows. Martinez et al. [11] reported that the DMI decreased when cows were fed a 60% high-forage total mixed ration (**TMR**) compared with a lower-forage TMR with similar milk production. Controlled research studies and field experiences have concluded that it is possible to maintain production when utilizing high-forage rations as long as consistent, highly digestible forages are fed [13,14]. Chase and Grant [13] demonstrated that dairy herds producing greater than 36 kg of milk were fed rations containing more than 70% forage. In addition, high-forage rations are beneficial in numerous ways, including reduced feed costs, increased cow health, rumen homeostasis, and improved nutrient management [13].

Buxton [15] reported that the most important factor influencing forage digestibility is the herbage stage of maturity. However, grass and legume forages accumulate sugars and starch from sunrise to sundown [16]. Thus, shifting cutting from morning to afternoon has demonstrated increased nonstructural carbohydrate concentrations [17,18,19]. Forages produced with higher nonstructural carbohydrate concentrations resulted in increased milk production, milk components, and dry matter intakes across several studies conducted by Brito’s research group [16,20,21,22]. In contrast with the harvest timing, limited field observations (Ag spectrum and internal data) evaluating specific soil and crop additive (**SCA**) crop production programs demonstrated an impact on producing forages that have greater nutrient digestibility. These digestibility improvements were achieved via soil-based saturation amendments, biostimulates, and foliar nutrient applications. The SCA agronomy programs used could improve plant health and growth characteristics. Thus, SCA utilization for crop production (focusing on pH, soil organic matter, and base saturation levels) in conjunction with agronomic management (including fertilizer application) programs for forage production, theoretically, would improve forage nutrient digestibility. Improved forage nutrient digestibility would increase the forage’s economic value, along with allowing for greater forage inclusion rates in rations, while still meeting the National Research Council’s (**NRC**; [23]) nutrient requirements for high-producing dairy cows. To our knowledge, no scientific reports exist in the literature evaluating forage production via soil and agronomy crop production programs and their impact on the milk production of lactating dairy cows.

The study objective was to evaluate the lactational performance of peak-lactation dairy cows fed forages (corn silage and alfalfa haylage) produced via the standard (control; **CON**) university (South Dakota State University; **SDSU**) soil and agronomy crop production program (minimal fertilization for only yield, with no focus on quality) compared with an SCA (pH and base saturation) and agronomy management (foliar application) program in the production of forages for formulating rations and feeding lactating dairy cows. The hypothesis was that an SCA crop production program for growing corn silage and alfalfa haylage would increase the nutrient digestibility to increase the energy and nutrient supply to the lactating dairy cows to meet their nutrient requirements for increased milk production.

## 2. Materials and Methods

### 2.1. Forage Production

In early Spring 2014, an existing 15.4 ha alfalfa field was split into 2 parcels of 9.3 ha for CON and 6.1 ha for the SCA agronomy program for growing alfalfa. The South Dakota State University (**SDSU;** Brookings, SD, USA) CON agronomy alfalfa protocol was followed, but no spring fertilization, according to soil tests, was required due to the pH being 7.8 and there being adequate P (145 ppm and 102 ppm for the alfalfa and corn fields, respectively) and K (186 and 205 ppm) concentrations. A foliar feed with boron and a generic insecticide were applied after the 1st, 2nd, and 3rd cutting. On 3 May 2014, for the SCA agronomy program, gypsum was applied at 981 kg/ha, potash at 112 kg/ha, and S at 75 kg/ha. On 14 May 2014, following alfalfa green-up, a foliar application of Kick-Off at 3.4 kg/ha, GroZyme at 1096 mL/ha, and Blitz at 439 mL/ha was applied (Ag Spectrum, Dewitt, IA, USA).

First-cutting alfalfa was harvested as alfalfa haylage (New Holland Chopper, CNH Industrial, New Holland, PA, USA) on 28 May 2014, with each loaded forage wagon weighed on a platform scale (SDSU Feed Mill, Brookings, SD, USA) and ensiled in a 2.4 m × 76.2 m Ag Bag (Ag-Bag by RCI, Mayville, WI, USA) with Silo-King (a silage inoculant containing lactic acid bacteria, enzymes, antioxidants, and a mold inhibitor; Agri-King, Inc. Fulton, IL, USA) applied at a rate of 0.5 kg/t on an “As Is” basis.

After each alfalfa cutting upon green-up, foliar applications were applied on 10 June, 8 July, and 4 August 2014. Second-cutting alfalfa haylage was harvested on 25 June 2014, treated with Silo-King at 0.5 kg/t, and ensiled in another Ag-Bag (RCI, Mayville, WI, USA). Due to the 1st and 2nd cuttings supplying ample tonnage for a lactating dairy cow feeding trial, the 3rd and 4th cuttings were harvested on 22 July 2014 and 26 August 2014 as alfalfa baleage, weighed, and shipped off to other SDSU livestock units. The days between cuttings were 30, 28, and 33, respectively. During this alfalfa growing and harvesting season, no manure was applied to the field between cuttings.

In early Spring 2014, a 13.4 ha field was split into 2 parcels, with 1 being approximately 9.3 ha for CON corn silage production and the 2nd parcel being approximately 5.3 ha for producing corn silage via the SCCA agronomy corn silage program. The field was fully tilled, and the CON agronomy program consisted of no supplemental fertilizer due to soil tests. During this corn silage growing and harvesting season, no manure was applied to the field. After planting, a pre-emergence herbicide, Harness Xtra (Bayer, Crop Science Division, Institute, WV, USA), was applied, and 89 mL of Callisto (Syngenta, Wilmington, DE, USA) and 946 mL of Powermax (Bayer, Crop Science Division, Institute, WV, USA) were sprayed before canopy as post-emergence herbicides. For the SCA agronomy crop production program, preplant gypsum was broadcast at 980 kg/ha, potash at 112 kg/ha, Blitz at 439 mL/ha, and GroZyme at 1096 mL/ha (Ag Spectrum, DeWitt, IA, USA). On 21 May 2014, the seed corn hybrid MC527 (Masters Choice, Anna, IL, USA) was planted at 79,000 kernels/ha, and CleanStart was applied at 32 L/ha, GroZyme at 292 mL/ha, Kick-Off at 5.36 kg/ha (Ag Spectrum, DeWitt, IA, USA), liquid potassium at 18 kg/ha, N at 209 kg/ha, and S at 29 kg/ha, which were side-dressed at planting. On 10 June 2014, a foliar application of GlyCure at 4.7 l/ha, GroZyme at 585 mL/ha, PT-21 at 37 l/ha, and Score at 5.6 kg/ha was applied (Ag Spectrum, DeWitt, IA, USA).

The corn was harvested as corn silage via a New Holland self-propelled chopper with a kernel processor (CNH Industrial, New Holland, PA, USA) on 7 October 2014. The corn silage was weighed, treated with Silo-King (Silage Inoculant, Agri-King, Inc. Fulton, IL, USA) at a rate of 0.5 kg/t on an “As Is” basis using a Gandy applicator (Gandy, Owatonna, MN, USA) mounted on an Ag Bagger, and ensiled in a 2.4 m × 76.2 m Ag Bag (Ag-Bag by RCI, Mayville, WI, USA).

### 2.2. Product Descriptions

Blitz (Ag Spectrum, DeWitt, IA, USA) is a surfactant/penetrant used to move GroZyme (Ag Spectrum, DeWitt, IA, USA) into a soil solution. CleanStart (Ag Spectrum, DeWitt, IA, USA) is derived from ammonia, urea, orthophosphoric acid, and potassium hydroxide to be a highly available phosphorus source. GroZyme (Ag Spectrum, DeWitt, IA, USA) is a biostimulant product produced via the microbial fermentation of a proprietary mix of organic cereal grains inoculated with specific bacterial cultures used to increase soil microbial activity and improve plant nutrient mobility. Soil applications of GroZyme (Ag Spectrum, DeWitt, IA, USA) have been reported to enhance soil microbial activity and nitrogen transformations [24,25]. Kick-Off (Ag Spectrum, DeWitt, IA, USA) is a micronutrient mix of Fe, Mn, Cu, and Zn predominantly derived from nitrate sources with additional surfactants and stabilizers for use on plants during the vegetative development stages. GlyCure (Ag Spectrum, DeWitt, IA, USA) is a micronutrient package used to resupply micronutrients in crops after exposure to glyphosate. PT-21 (Ag Spectrum, DeWitt, IA, USA) is a urea nitrogen source used to supply N and improve other minerals’ movement across the cuticle layer. Score (Ag Spectrum, DeWitt, IA, USA) is a micronutrient mixture for use in plants either entering or in the reproductive development stages. The elemental compositions of all spray applications are provided in Tian et al.’s study [26]. These are all proprietary products formulated, produced, and marketed by Ag Spectrum (DeWitt, IA, USA).

### 2.3. Lactation Experiment

The research lactation experiment was conducted at the SDSU Dairy Research and Training Facility (**DRTF**; Brookings, SD, USA) from November 2014 to June 2015, following the guidelines that became the 4th edition of the *Guide for the Care and Use of Agricultural Animals in Research and Teaching* [27]. All lactating dairy cows were cared for and managed according to the SDSU Institutional Animal Care and Use Committee recommendations. The cows were housed in a curtain-sided free-stall barn equipped with free access to water, with Calan feeding doors and feed boxes (American Calan Inc., Northwood, NH, USA). A total of 30 peak-lactation (58 days in milk (**DIM**) ± 2.9, 38.9 kg milk ± 7.6, and 630 kg body weight (**BW**) ± 97.7) Holstein dairy cows (8 primiparous and 22 multiparous), were blocked by milk yield, DIM, and parity, and randomly assigned to 1 of 2 TMR treatments using a randomized complete block design (**RCBD**) with a pretreatment covariate. The experimental period was a continuous 13 wk experiment, with the first 7 d for diet adaptation (using a SCON ration fed for the covariate period) and adjustment, followed by 84 d (12 wk) of data collection.

The treatments were as follows: (1) for CON, forages (61.6:38.4 corn silage–alfalfa haylage mixed at 65:35 forage–concentrate in the TMR) were formulated using 1st-cutting alfalfa haylage and corn silage produced via the CON SDSU agronomy program; (2) for SCA, forages (same ratios) were formulated using 1st-cutting SCA alfalfa haylage and corn silage. The ingredient composition of the grain mix was similar between both treatments and was mixed at the SDSU Feed Mill and delivered to the DRTF approximately every 2 wk (Table 1). All cows were fed the CON TMR during the 7 d covariate period, followed by 12 weeks of data collection when the CON and SCA TMRs were fed. All TMRs were prepared and delivered using a Calan Super Data Ranger (American Calan, Inc., Northwood, NH, USA). The TMR was formulated using NDS Professional (Nutritional Dynamic System, Emilia, Italy), a Cornell Net Carbohydrate and Protein System (**CNCPS**)-based platform for ruminant ration formulation and evaluation to predict the lactating dairy cow performance for a 616 kg Holstein cow producing 38.6 kg/d of milk, 3.75% fat, and 3.36% crude (total) protein, consuming approximately 24.2 kg of DM/d. A post hoc power and sample size analysis (SAS version 9.4, Cary, NC, USA) with an SEM of 1.70 indicated that 14 cows/treatment were needed to detect a 7% increase in milk production at greater than 90% power without the use of a covariate.

The cows were milked 3 times daily (at 0600, 1400, and 2100 h) and fed individually (Calan Feeding Doors, American Calan, Inc., Northwood, NH, USA) once daily (at 0700 h) via ad libitum intake with 5 to 10% orts. The total daily feed offerings were adjusted based on the previous 24 h intake to achieve approximately 5% refusals. The amounts fed and orts were recorded daily. The forages were tested weekly for dry matter (**DM**), and forage adjustments were made for changes in the DM concentrations. The health status of each animal was evaluated daily, and all other bedding, cow monitoring, and manure scraping followed standard DTRF operating procedures supervised by the SDSU attending veterinarian (Dr. Michelle Mucciante).

### 2.4. Data and Sample Collection

An approximately 500 g aliquot of forage was collected from every load of alfalfa haylage at each cutting or corn silage during harvest and composited into 1 large sample per cutting or crop. These samples were thoroughly mixed, subsampled, dried for 48 h at 55 °C, and ground through a 4 mm screen (Wiley mill, Arthur H. Thomas Co., Philadelphia, PA, USA), followed by grinding through a 1 mm screen using an ultracentrifuge mill (Brinkman Instruments Co., Westbury, NY, USA), and submitted to Analab (Fulton, IL, USA) for nutrient analyses.

Prior to the start of the experiment, the forages were sampled, and the nutrients were analyzed after ensiling, followed by the TMR formulation using actual forage and feed ingredient nutrient concentrations. The dry matter compositions of the forages were determined weekly by drying them in a 105 °C oven (Despatch LEBI-75, Despatch Industries, Minneapolis, MN, USA) for 24 h, and the feed sheets were adjusted accordingly to maintain the ingredients at constant DM concentrations. Samples of the grain mix, individual forages, and TMR were collected and frozen (−20 °C) weekly for future analysis. The daily intake was calculated from the TMR offered and refusals, with the DM intake (**DMI**) being calculated by correcting the daily intake with the DM concentration. Refusals were assumed to be representative of the TMR fed due to achieving 5 to 10% feed refusals.

The milk production was recorded electronically (DeLaval-ALPRO, Kansas City, MO, USA) at each individual milking and saved daily to a Universal Serial Bus flash drive. Three milk samples were collected at each of the daily milkings, once each wk, from each individual cow. One set of milk samples was composited by day on a weighted basis, proportional to the milk production, and frozen at −20 °C for potential future protein fraction and/or fatty acid composition analyses. The other set of individual milk samples was sent to Dairy Herd Improvement Association Heart of America (Manhattan, KS, USA) for analyses of fat, protein, somatic cell counts (**SCCs**), lactose, and milk urea nitrogen (**MUN**) using Association of Official Analytical Chemists (**AOAC**) International-approved [28] procedures.

Body weights (**BWs**) were electronically collected using a digital livestock scale (AWB-5K-SYS, Triner Scale and Manufacturing Company, Inc., Olive Branch, MS, USA), approximately 3 h after feeding on Thursday of each week. Body condition scores were determined weekly by the same 3 individuals on a scale of 1 to 5, with 1 as emaciated and 5 as obese [29], using Edmondson et al.’s [30] scoring chart, approximately 3 h after feeding at the start of this study (covariate) and each week. The scores of the 3 individuals were averaged.

Rumen fluid samples were collected on Thursday of wk 4, 8, and 12 at approximately 3 h after feeding via an esophageal tube attached to a hand-operated pump. The first 100 mL of rumen fluid was discarded to minimize saliva contamination. After collection, the rumen fluid was mixed thoroughly, and the pH was immediately measured using an electronic pH meter (Corning 350, Corning Inc., Corning, NY, USA). If the rumen fluid collected had a pH of >7.0, the rumen fluid was discarded, and additional rumen fluid was collected to ensure minimal saliva contamination. Two 10 mL samples of rumen fluid were collected, where one 10 mL sample was added to a vial containing 200 µL of 50% (vol/vol) H_2_SO_4_ for later determination of NH_3_-N, and the other 10 mL sample was added to a vial containing 2 mL of 25% (wt/vol) meta-phosphoric acid for later volatile fatty acid (**VFA**) determination. After the sample collection and preparation, the rumen fluid samples were immediately stored at −20 °C.

One 6 mL coccygeal artery blood sample, using a vacutainer tube containing sodium fluoride and a 20 guage 0.9 mm × 25 mm needle (Beckton Dickinson Vacutainer Systems, Rutherford, NJ, USA), was also collected on Thursday of wk 4, 8, and 12 at 3 h after feeding for later analysis of blood glucose concentrations. Fecal grab samples were collected during wk 4, 8, and 12 every 8 h for 3 d, with a forward advancement of 2 h daily to account for diurnal variation. The samples were composited by cow and stored frozen at −20 °C.

### 2.5. Laboratory Analysis

At the end of the experiment, the feed samples (TMRs, concentrate mixes, and individual ingredients) were thawed and composited by month and dried at 55 °C for 48 h in a forced-air oven (Style V-23, Despatch Oven Co., Minneapolis, MN, USA). The feed sample composites were ground through a 4 mm screen (Wiley mill, Arthur H. Thomas Co., Philadelphia, PA, USA) and then further ground through a 1 mm screen using an ultracentrifuge mill (Brinkman Instruments Co., Westbury, NY, USA) before being sent to Analab (Fulton, IL, USA) for DM and nutrient analyses. The feed samples were analyzed for their nutrient concentrations following standard AOAC International methods [28], including DM (935.29), crude protein (**CP;** 990.03), neutral detergent fiber (**NDF**) with amylase (2002.04), acid detergent fiber (**ADF**; 973.18), ADF-insoluble nitrogen (**ADIN**; 973.18 and 976.06), NDF-insoluble protein (**NDIP;** 2002.04 without sulfite and 976.06), lignin (973.18), ash (942.05), Ca (985.01), P (985.01), Mg (985.01), Na (985.01), Cl (915.01), S (923.01), Fe (985.01), Cu (985.01), Zn (985.01), K (985.01), Mn (985.01), and pH (981.12). The remaining nutrient concentrations were measured using the following methods: soluble protein (**SP**) [31], starch [32], oil [33], in vitro dry matter digestibility (**IVDMD**) (24 h ruminal and 24 h enzymatic digestion using the Kansas State Buffer) [34], neutral detergent fiber digestibility (**NDFD**) ([35]; incubation for 30 h using the Kansas State Buffer [31]), ammonia–nitrogen (**NH_3_-N**; the United States Environmental Protection Agency, 1993, method 351.2, and the International Organization for Standardization, 2013, method 11732), lactic acid [36], acetic acid [37], non-fiber carbohydrate (**NFC;** [23]), net energy of lactation (**NE_L_**; [23]), relative forage quality (**RFQ;** [38]), and sugar [39]. Hemicellulose (**HC**) was calculated as HC = NDF – ADF.

The milk fat, protein, and lactose were analyzed using near-infrared spectroscopy (Bentley 2000 Mid-Infrared Milk Analyzer, Bentley Instruments, Chaska, MN, USA). The milk urea nitrogen concentrations were determined using a chemical methodology based on a modified Berthelot reaction (ChemSpec 150 Analyzer, Bentley Instruments, Chaska, MN, USA; [40]). The somatic cell counts were determined using a flow cytometer laser (Somacount 500, Bentley Instruments, Chaska, MN, USA; [28]). The somatic cell counts were converted to a linear somatic cell score (**SCS**) using the following equation: [(ln(SCC/100))/0.693147] + 3, as described by Spaniol et al. [41]. The fat-corrected milk (**FCM**; 3.5%) was calculated using the following equation: (0.432 × kg milk) + (16.216 × kg fat). The energy-corrected milk (**ECM**) was calculated using the following equation: (0.327 × kg milk) + (12.95 × kg fat) + (7.65 × kg protein), as described by Orth [42]. The fat- and protein-corrected milk (**FPCM**) was calculated according to the IDF [43] equation: FPCM = Milk, kg/d × ((0.1226 × fat, %) + (0.0776 × protein, %) + 0.2534).

The rumen fluid samples were thawed and centrifuged at 30,000× *g* for 20 min at 20 °C (Eppendorf 5403, Eppendorf North America, Hauppauge, NY, USA). Rumen fluid samples acidified with 50% (vol/vol) H_2_SO_4_ were analyzed for ruminal NH_3_-N using the Chaney and Marbach [44] procedures. Ruminal fluid samples acidified with 25% (wt/vol) meta-phosphoric acid were prepared according to Erwin et al. [45] and analyzed for VFA concentrations using an automated gas–liquid chromatograph (model 6890, Hewlett-Packard, Palo Alto, CA, USA) with a flame ionization detector. Once prepared, 1 µL of each prepared sample was injected with a split ratio of 30:1 at the injection port (250 °C). The VFAs were separated in a capillary column (15 m × 0.25 mm i.d.; Nukol, 17926–01C, Supelco Inc., Bellefonte, PA, USA) with a flow rate of 1.3 mL/min of He, using 2-ethylbutyrate as an internal standard. The column and detector temperatures were maintained at 140 °C and 250 °C, respectively. Blood plasma taken 3 h after feeding was analyzed for its glucose concentration via a colorimetric enzymatic kit (Liquid Glucose (Oxidase) Reagent Set; Pointe Scientific, Inc., Canton, MI, USA).

Individual (cow) fecal composite samples were shipped frozen in insulated shippers to Analab Laboratory (Fulton, IL, USA) for nutrient analysis. The fecal samples were analyzed using the following Association of Official Analytical Chemists International [28] methods: DM (935.29), CP (990.03), ADF (973.18), and NDF (2002.04). Starch was measured using the Hall [32] method. Acid-insoluble ash (**AIA**) was analyzed in both the feed and feces as an internal digestibility marker [46]. The nutrient digestibility was calculated as follows: digestibility, % = 100 − (100 × (AIA TMR concentration/AIA fecal concentration) × (nutrient fecal concentration/nutrient TMR concentration)).

### 2.6. Statistical Analysis

All data were checked for normality and outliers using the univariate procedure of SAS (version 9.4, SAS Institute Inc., Cary, NC, USA) before any statistical analyses were conducted. Box and whisker plots and the Shapiro–Wilk test were used to verify that the data were normally distributed (*p* > 0.15). All data were then subjected to a least-squares analysis of variance (ANOVA) for an RCBD [47] with a covariate using the PROC MIXED procedure of SAS (Version 9.4, SAS Institute, Inc., Cary, NC, USA), consisting of 2 treatments and 15 blocks. The statistical model used was as follows:

Y_ijkl_ = µ + Block_i_ + P_j_(Block_i_) + T_k_ + W_l_ + (T_j_ × W_k_) + Cov + Cow_m_(T_k_) + e_ijkl_

where Y_ijkl_ = the dependent variable, µ = the overall mean, Block_i_ = replication or block, P_j_(Block_i_) = parity (primiparous vs. multiparous) nested within replication, T_k_ = the treatment, W_l_ = the experimental week, T_k_ × W_l_ = the treatment-by-week interaction, Cov = the pretreatment covariate for the appropriate variables, Cow_m_(T_k_) = cow nested within treatment, and e_ijkl_ = the residual random error. The cows were blocked according to the calving date and parity (4 primiparous vs. 11 multiparous/treatment). Due to parity being nested within Rep and the use of a covariate, Rep was found to be nonsignificant, and, therefore, replication was deleted from the statistical model, but parity remained in the model. The individual cow data from the covariate week (the week before the experimental start) were included in the model. The treatment, week, and treatment × week interactions were considered fixed effects, with cow nested within treatment as a random effect. The experimental week was considered a repeated measurement in time, with an autoregressive covariance structure. The somatic cell counts were log_10_ base-transformed prior to the statistical analyses. The least-squares covariate-adjusted means were separated by the PDIFF statement. The PDIFF statement is the least-significant difference method, which was used to compare the treatment means when the ANOVA F-test was significant. All results are reported as least-square means. Differences between treatments were considered significant at *p* < 0.05 and trends at 0.05 < *p* ≤ 0.10.

## 3. Results

### 3.1. Forage Production and Quality

Because this study’s focus was on producing forages for a feeding trial, no opportunity existed to replicate the forage crop production component of this experiment by using field plots. However, field yield and quality data were still obtained, although these data could not be statistically analyzed. First, the 2014 crop growing season was cooler and wetter compared with the 30-year average for Brookings, SD, USA (Table 2). Climatic conditions are known to impact forage yield and digestibility [48]. However, since the forages were grown side by side in the same fields with the same weather conditions and soil types, the field differences should have been minimal to nonexistent, except for any weather conditions impacting the forage yield and digestibility in addition to the agronomy applications.

Growing alfalfa using an SCA crop production program resulted in an improvement (5.38% and 0.80% for cuttings 1 and 2, respectively) in the DM yield compared with the CON SDSU agronomy program (Table 3). During the foliar application after harvesting the second alfalfa cutting during green-up for growing the third alfalfa cutting, the custom applicator’s sprayer tank apparently was not thoroughly cleaned. The third alfalfa crop was visually observed to be severely stressed but not killed. The previous crop was sprayed with the contracted custom sprayer equipment, and the company used a glyphosate herbicide in the tank. The apparent glyphosate residual in the tank severely damaged the third- and fourth-cutting alfalfa yields for the SCA-grown alfalfa compared with the CON-grown alfalfa (Table 3). Glyphosate is toxic to non-genetically modified alfalfa, and the alfalfa recovery rate has been demonstrated to be slow and incomplete [49]. Therefore, the yield data for the third- and fourth-cutting alfalfa were severely compromised due to uncontrollable human circumstances.

The crude protein concentrations were approximately 10% numerically greater, and the digestibility (NDFD and IVDMD) measurements were approximately 13.0 and 3.8%, and 4.2 and 6.3%, numerically lower for the first- and second-cutting alfalfa haylage, respectively, produced via the SCA crop production program compared with the CON-grown alfalfa haylage (Table 4). The CP and IVDMD were numerically greater for the glyphosate-damaged third- and fourth-cutting alfalfa compared with the CON-grown alfalfa. However, glyphosate-stunted alfalfa growth would likely increase the leaf-to-stem ratio, which would impact the measurements of CP and IVDMD concentrations. Lissbrant et al. [50] reported that higher DM digestibility was associated with lower alfalfa yields. It is speculated that glyphosate-stunted alfalfa growth could reduce the onset of maturity, which would improve DM digestibility [15]. The first cutting was the only alfalfa haylage targeted for use in the lactation trial. The remaining cuttings were used for general herd feeding or shipped to other SDSU research units.

The reason for selecting the Masters Choice seed corn hybrid MC527 to produce the corn silage was based on our earlier work, demonstrating a greater canopy and softer starch structure that resulted in similar milk production with lower total TMR starch concentrations [6,51]. The DM yield difference was only 2.2% between the CON-grown corn silage and the SCA-grown corn silage. The corn silage nutrient compositions produced via both agronomy programs were numerically similar, except for a numerical advantage (2.2%) in the starch concentration for the SCA crop production program compared with the CON crop production program. The initial measurements of the corn silage’s NDF digestibility based on the harvested samples (pre-ensiling) were numerically (approximately 6.8%) in favor of the CON treatment compared with the SCA agronomy program.

### 3.2. Lactation Total Mixed Rations

The forage composition of the lactation TMR fed to both treatment groups consisted of 23.4% first-cutting alfalfa haylage and 37.6% corn silage (DM basis). The analyzed TMR nutrient concentrations were similar (*p* > 0.05; Table 5) for lactating dairy cows fed both treatments. The TMR nutrient concentrations met formulation expectations and would meet or exceed the nutrient requirements of peak-producing dairy cows [23]. In contrast with the initial forage nutrient concentrations (Table 4), the measurements of NDFD and IVDMD indicated a numerically greater (*p* < 0.14) nutrient digestibility for the SCA TMR compared with the CON TMR (Table 5) after ensiling. It is plausible that some ensiling effects may have affected digestibility, i.e., acid hydrolysis.

### 3.3. Lactational Performance

The treatment-by-week interactions for all parameters were similar (*p* > 0.10) for cows fed both treatments. Milk production was increased (*p* < 0.04) by approximately 13.2% for cows fed SCA-grown forages compared with cows fed CON-grown forages (Table 5). The greatest change in milk production occurred during the first week of the experiment when lactating dairy cows were switched from the CON TMR-grown forages during the covariate period to the SCA TMR-grown forages (approximately 2.0 kg/d). In addition, cows fed the SCA-grown forages were more persistent in maintaining milk production throughout the experimental period compared with cows fed the CON-grown forages. These data demonstrate that improving forage digestibility through an agronomy crop production program can increase milk production. In theory, a higher forage component in the ration could have been fed to maintain the same milk production while reducing the ration cost. Llamas-Lamas and Combs [10] reported that more-digestible forages can be consumed in greater amounts compared with less-digestible forages. Meanwhile, Cherney et al. [14] reported that feeding a moderately high-forage ration could maintain milk production while reducing feed costs. In contrast, Gadeken and Casper [5] fed a highly digestible forage at 80% of the ration, but the digestibility was not sufficiently high to meet the nutrient requirements, thereby resulting in reduced milk production in the late-lactation dairy cows. These data and reported studies demonstrate that forage inclusion amounts in rations should be based on forage digestibility measurements.

The milk component percentages were similar (*p* > 0.20) for lactating dairy cows fed using both agronomy forage production programs. The numerically lower fat percentages for cows fed SCA-grown forages compared with cows fed CON-grown forages resulted in similar (*p* > 0.26) milk fat yields, but the production of other milk components, i.e., protein, lactose, SNF, and total solids, was greater (*p* < 0.05) for cows fed SCA-grown forages compared with cows fed CON-grown forages. The greater milk production for lactating dairy cows fed SCA-grown forages with similar (*p* > 0.20) milk component concentrations resulted in a trend (*p* < 0.09) and a greater (*p* < 0.05) production of 4% FCM and ECM, respectively, compared with lactating dairy cows fed CON-grown forages. The somatic cell counts (*p* > 0.63) and MUN (*p* > 0.37) were similar for cows fed using both agronomy forage programs. Gadeken and Casper [5] reported no differences in milk composition, which agreed with Martinez et al. [11] when feeding a 60% forage TMR compared with an 80% forage TMR. The forage grown in the SCA crop production program not only increased milk production but also maintained the milk composition, resulting in increased milk component production as well.

The peak-lactation dairy cows fed both grown forage treatments were similar (*p* > 0.14) in their DMI; initial, final, and mean BWs; ADG; DMI as a % of BW; and BCS. The milk production increase combined with the similar DMI resulted in an improved feed efficiency when expressed as milk/DMI (*p* < 0.01), or FCM/DMI (*p* < 0.05), for lactating dairy cows fed SCA compared with lactating dairy cows fed CON, while the ECM/DMI was similar (*p* > 0.13). The improvements in the feed efficiency indicate a greater supply of available energy and nutrients in the SCA-grown forages compared with the CON-grown forages. The numerical improvements in the NDFD and IVDMD (Table 5) confirm that more nutrients were available for milk production from the same amount of DM (Table 6). Casper and Mertens [7] reported that the greatest factor affecting energy and nutrient digestibility was digestible fiber based on an analysis of the Energy Metabolism Unit database. Dado and Allen [52] reported an increased DMI and milk production using similar NDF ration concentrations but differing NDFD. Gadeken and Casper [5] reported a similar feed efficiency when feeding 60 and 80% forage TMRs to late-lactation dairy cows, but Martinez et al. [9] reported an improved FE when feeding a higher-forage ration. Casper [2] reported that preliminary research suggested the maximum intake of indigestible fiber was 0.45% of BW, which determines the maximum amount of NDF that a dairy cow can consume. Thus, cows fed the SCA-grown forages consumed more digestible fiber. Previous studies [53,54] reported that highly digestible forages can be fed at a greater ration percentage while maintaining DMI, milk production, and feed efficiency.

### 3.4. Ruminal Fermentation

The ruminal pH values were similar (*p* > 0.22) for lactating dairy cows fed both grown forage treatments (Table 7). In contrast, Cherney et al. [14] reported a lower ruminal pH when cows were fed brown midrib corn silage and conventional corn silage compared with leafy hybrid corn silage. The differences could be the result of greater ruminal fiber and starch digestion. In agreement, Dado and Allen [52] reported a similar ruminal pH when feeding alfalfa silage with different NDF digestibilities. Arndt et al. [55] reported a similar ruminal pH when altering the alfalfa silage-to-corn silage ratio.

The serum blood glucose and ruminal ammonia concentrations were similar (*p* > 0.63) for cows fed both forage treatments. Forage digestibility and the forage–concentrate ratio have been reported [6,53] to have a minimal impact on ruminal ammonia N concentrations. Arndt et al. [55] reported that increasing the alfalfa silage-to-corn silage ratio resulted in greater ruminal ammonia concentrations.

Lactating dairy cows fed via both forage-growing programs showed similar (*p* > 0.57) total ruminal VFA concentrations. The molar acetate concentrations were similar (*p* > 0.10) for cows fed both forage programs; however, cows fed SCA-grown forages demonstrated a tendency (*p* < 0.10) for greater molar propionate concentrations compared with cows fed CON-grown forages. Dado and Allen [52] reported that cows fed alfalfa silage with similar NDF concentrations but differing NDF digestibilities demonstrated greater ruminal propionate concentrations.

The shift for lactating dairy cows to greater ruminal molar propionate concentrations when fed SCA-grown forages could explain the increase in lactose synthesis. Propionate is the precursor for liver glucose synthesis, and glucose is readily taken up by the mammary glands for lactose synthesis [56,57]. Greater lactose synthesis would explain the increase in milk production since lactose is the major osmotic regulator of milk production. Lactose is the primary osmoregulator of milk synthesis [56,57,58].

The molar butyrate concentrations were lower (*p* < 0.01) for cows fed SCA-grown forages compared with cows fed CON-grown forages. The molar concentrations of the remaining VFAs were similar (*p* > 0.23) for cows fed via both forage treatment programs. The acetate-to-propionate ratio was similar (*p* > 0.24) for cows fed both forage programs, while the acetate + butyrate-to-propionate ratio was numerically (*p* < 0.13) lower for cows fed SCA-grown forages compared with cows fed CON-grown forages, which may be related to the numerically lower milk fat percentages for cows fed SCA-grown forages (Table 6). Acetate and butyrate are both precursors for milk fat synthesis by the mammary glands [57,58]. Acharya and Casper [6], when feeding corn silage with greater NDFD, reported greater acetate/propionate and acetate + butyrate/propionate ratios in cows at peak lactation, while Dado and Allen [52] reported similar ratios.

### 3.5. Total-Tract Nutrient Digestibility

Peak-lactation dairy cows fed both crop program-grown forages showed similar apparent total-tract DM (*p* > 0.69) and CP (*p* > 0.20) digestibilities (Table 8). However, the apparent total-tract NDF and ADF digestibilities were approximately 12.5% greater (*p* < 0.03) for cows fed SCA-grown forages compared with cows fed CON-grown forages. In addition, the starch digestibility tended (*p* < 0.06) to be greater for cows fed SCA-grown forages compared with cows fed CON-grown forages. The increases in fiber and starch digestion provided additional energy and nutrients to support the increase in milk production (Table 6). Martinez et al. [11] reported that feeding a 60% forage ration reported a weak positive trend in DM digestibility (*p* < 0.13) compared with feeding a 50% forage ration, while no differences were observed in CP and NDF digestibilities. Dado and Allen [52] reported greater DM, NDF, hemicellulose, and cellulose digestibilities when feeding alfalfa silage with similar NDF contents but greater NDF digestibility.

## 4. Plausible Explanations

Laboratory assays, even though extensive, are still not able to measure all potential parameters that may be important to lactating dairy cows for nutrient digestion and absorption. Therefore, the lactating dairy cows fed SCA-grown forages produced more milk than would have been predicted by the initial forage nutrient assays. The improvements in the apparent total-tract NDF, ADF, and starch digestibilities would suggest that these dairy cows were digesting, extracting, and absorbing more energy and nutrients, which was not predicted by the initial forage nutrient assays. It is not known what forage nutrient parameters the laboratory assays should/could be measuring. However, the dairy cows were apparently digesting and extracting more nutrients to supply the additional nutrients required for an increase of 4.3 kg/d of milk.

The speculation is that the SCA forage program altered the structure of the lignin–fiber relationship in the growing plant, which allowed for more fiber digestion. We speculate that the SCA agronomy program could modify the ratio of ether to ester linkages in the forage [59]. Altering the ratio of ether to ester linkages between the fiber and lignin structure of the plant has the potential to improve nutrient digestibility.

With regard to the starch digestibility improvements, the speculation is that the ratio of amylose to amylopectin and/or the amount of prolamins could have been altered in the growing plant. Further research work is needed to confirm or refute these speculations about altering the fiber and starch structures. The limitations of this study are that changes in fiber and starch digestion could have altered the feeding behavior. In addition, these alterations more than likely altered the ruminal microbial community, which could be a future research area.

The nutrient digestibility calculations by combining the DMI, ration composition, and digestibility indicated that the amounts of total-tract digestion of NDF (3.23 and 3.32 kg/d), ADF (2.09 and 2.12 kg/d), and starch (6.06 and 5.91 kg/d) would not completely explain the increase in milk production for cows fed SCA-grown forages. In addition, the shift in ruminal fermentation helps explain the additional increase in milk production, i.e., propionate to glucose to lactose, but the total VFA concentrations were similar. However, ruminal VFA concentrations are not highly correlated with ruminal VFA production and absorption [60]. In addition, these calculations suggest that future research should evaluate ruminal versus total-tract digestion to elucidate the responses. There is limited published research in this area. Further research is warranted to elucidate the mechanism of the SCA agronomy program producing forages that are greater in nutrient availability to meet the nutrient requirements of high-producing lactating dairy cows.

The hypothesis that the implementation of an SCA agronomy program for growing forages by adjusting the soil base saturation, combined with foliar nutrition application, produced higher digestible forages that led to greater milk production, milk component production, fiber digestibility, and feed efficiency when lactating dairy cows were fed a higher-forage ration at peak lactation was confirmed. The milk production increase can partially be explained by the increase in the total-tract NDF and ADF digestibility of the SCA-grown forages. High-quality forage, supplemented at a high level, can increase feed efficiency, which is explained by the increases in milk production with a maintained DMI. The increases in NDF and ADF digestibility in the SCA forages support the explanation for this increase in milk yield and animal performance. The rate of alfalfa haylage NDF digestion is speculated to be a contributing factor to the increase in lactational performance. However, this study demonstrated that high-forage rations based on locally produced highly digestible forage can be fed to reduce feed costs to maintain or increase lactation performance.

## 5. Conclusions

Based on the positive performance demonstrated in this experiment, additional research is warranted to further advance and strengthen these findings. To our knowledge, this is the first study to evaluate SCA agronomy crop production practices for impacting forage production, which subsequently impacts the lactational performance of dairy cows fed these forages. The initial forage nutrient analyses indicated very little benefit to feeding these forages to lactating dairy cows. However, feeding these forages produced by the SCA agronomy crop production program resulted in a 13.5% improvement in milk production and an approximately 17% improvement in feed efficiency compared with cows fed CON forages with a similar DMI. The milk production response remains a critical measure of forage quality in dairy nutrition research.

## Figures and Tables

**Table 1:** Ingredient compositions of total mixed rations (TMRs) based on control (CON) or soil and crop additives (SCA) agronomy crop production program-grown forages fed to lactating dairy cows.

	TMR	
Ingredient	CON	SCA
	----------------- (% of DM) -----------------	
Corn silage, CON	37.6	-------
Alfalfa haylage, CON	23.4	-------
Corn silage, SCA	-------	37.6
Alfalfa haylage, SCA	-------	23.4
Ground corn, fine	15.7	15.7
Corn dried distillers grains	5.63	5.63
Whole cottonseed	5.63	5.63
Soybean meal, 47.5% CP	5.06	5.06
Expeller SBM/RP Lys ^1^	3.47	3.47
Limestone, 38% Ca	0.83	0.83
Salt, white	0.47	0.47
Sodium bicarbonate	0.41	0.41
Prilled fat ^2^	0.38	0.38
Dicalcium phosphate	0.28	0.28
Dried yeast culture ^3^	0.23	0.23
Dynamate	0.21	0.21
Trace mineral premix ^4^	0.19	0.19
RP lysine ^5^	0.13	0.13
Potassium chloride, white	0.11	0.11
RP methionine ^6^	0.10	0.10
Urea, 281 CP	0.09	0.09
Magnesium oxide	0.08	0.08
Monensin ^7^, 198.4 g/kg	0.01	0.01

^1^ Soybest Pearl—expeller soybean meal with rumen-protected lysine, Grain States Soya, West Point, NE, USA; ^2^ Energy Booster 100, Milk Specialties Global, Eden Prairie, MN, USA; ^3^ dried yeast culture, Diamond V XP yeast, Cedar Rapids, IA, USA; ^4^ 10% magnesium, 2.5% zinc, 1.9% manganese, 325 mg/kg cobalt, 5830 mg/kg copper, 325 mg/kg iodine, 1515 mg/kg selenium, 544,320 IU/kg vitamin A, 186,850 IU/kg vitamin D, and 2180 IU/kg vitamin E; ^5^ rumen-protected lysines, LysiPEARL, Kemin Industries, Des Moines, IA, USA; ^6^ rumen-protected methionine, Mepron, Evonik Corporation, Kennesaw, GA, USA; ^7^ Monensin, Rumensin, Elanco Animal Health, Greenfield, IN, USA.

**Table 2:** Climatic conditions during 2014 growing season ^1^.

	Temperature, °C		Precipitation, mm	
Month	Mean	Deviation ^2^	Total	Deviation ^2^
April	1.5	−5.2	44.5	24.9
May	10.5	−2.8	52.3	13.4
June	16.7	−2.7	224.0	172.2
July	17.0	−4.7	61.2	27.2
August	11.3	−8.7	73.7	41.2
September	11.3	−4.3	50.8	27.2
October	4.7	−3.1	16.8	8.9

^1^ Data collected from South Dakota State University weather station; ^2^ deviation = actual—minus 30 year monthly average.

**Table 3:** Forage yields of control (CON) and soil and crop additive (SCA) crop production programs for growing forage.

	Forage Crop Program		
Forage	CON	SCA	Difference
Alfalfa			
1st cutting, Mg DM/ha	2.23	2.35	0.12
2nd cutting, Mg DM/ha	2.51	2.53	0.02
3rd cutting, Mg DM/ha	1.40	0.70	−0.62
4th cutting, Mg DM/ha	2.84	2.17	−0.67
SD	0.61	0.84	-----
Corn silage, Mg DM/ha	13.9	13.6	−0.3

**Table 4:** Pre-ensiling nutrient composition (% dry matter basis) for harvested alfalfa and corn silage forage quality grown via control (CON) and soil and crop additive (SCA) agronomy programs.

	Alfalfa, 1st Crop		Alfalfa, 2nd Crop		Alfalfa, 3rd Crop		Alfalfa, 4th Crop		Corn Silage	
Nutrient	CON	SCA	CON	SCA	CON	SCA	CON	SCA	CON	SCA
DM, %	49.0	46.8	44.6	43.3	84.4	87.0	88.1	86.4	44.5	46.1
CP	24.9	27.4	26.3	26.5	19.9	21.3	15.9	18.6	6.9	6.7
Fat	1.74	1.88	2.04	1.89	0.71	0.82	1.28	1.06	2.55	2.61
NDF	35.1	34.1	29.5	36.8	54.0	51.5	47.2	46.3	36.5	35.9
ADF	26.0	26.7	21.8	24.1	39.9	36.8	33.0	35.6	19.1	19.1
HC ^1^	9.1	7.4	7.7	12.7	14.1	14.7	14.2	10.7	17.4	16.8
Lignin	5.0	5.6	4.6	4.9	8.2	7.8	6.9	8.0	1.24	1.40
Starch	-----	-----	-----	-----	-----	-----	-----	-----	41.1	42.2
NFC	31.6	30.3	34.4	31.8	21.8	24.6	31.5	29.6	51.3	51.3
Ash	8.63	8.73	10.6	10.1	8.51	7.10	6.18	6.67	3.42	3.11
Ca	1.63	1.76	1.57	1.54	1.28	1.15	0.88	1.01	0.16	0.19
P	0.37	0.39	0.41	0.40	0.34	0.30	0.28	0.33	0.23	0.22
Mg	0.40	0.45	0.41	0.45	0.38	0.38	0.24	0.46	0.15	0.15
K	2.33	2.37	2.58	2.44	2.14	1.67	2.02	2.21	0.86	0.99
Cl	0.45	0.81	0.43	0.65	0.25	0.43	0.44	0.59	0.24	0.34
S	0.25	0.35	0.29	0.34	0.20	0.28	0.14	0.20	0.06	0.05
Na	0.08	0.09	0.12	0.15	0.09	0.13	0.10	0.15	0.01	0.01
NDFD ^2^	57.3	50.7	63.9	61.3	47.7	47.4	48.4	42.6	50.6	47.4
IVDMD ^3^	79.7	76.8	84.2	79.2	62.7	66.2	64.6	66.2	76.8	76.7

^1^ HC = hemicellulose, NDF—ADF; ^2^ NDFdD= 30 h neutral detergent fiber digestibility, % of NDF; ^3^ IVDMD = in vitro dry matter digestibility.

**Table 5:** Nutrient compositions of forages grown via control (CON) and soil and crop additive (SCA) agronomy programs and incorporated into a total mixed ration (TMR) fed to lactating dairy cows (%DM).

	TMR			
Nutrient	CON	SCA	SEM	*p*< ^1^
N	8	8	-----	-----
DM, %	56.6	56.8	0.84	0.90
CP, %	18.5	19.2	0.26	0.06
SP, % of CP ^2^	44.0	42.0	1.12	0.23
NDF	27.9	26.6	0.48	0.10
Hemicellulose	9.8	9.6	0.33	0.57
ADF	18.1	17.1	0.70	0.12
Cellulose	14.9	13.9	0.37	0.07
Lignin	3.25	3.15	0.18	0.61
Starch	25.9	26.3	0.68	0.68
NFC	44.4	44.6	0.79	0.91
Ash	6.39	6.77	0.21	0.21
NE_L_, Mcal/kg	1.80	1.83	0.01	0.13
Ca	0.85	0.89	0.02	0.84
P	0.40	0.41	0.01	0.35
Mg	0.28	0.28	0.01	0.89
K	1.51	1.59	0.03	0.12
S	0.27	0.30	0.01	0.04
Na	0.3	0.32	0.01	0.37
Cl	0.62	0.71	0.01	0.01
NDFD, 30 h ^3^	58.5	60.4	1.30	0.25
IVDMD, % ^4^	82.7	84.1	0.77	0.14

^1^ Probability of *F*-test for treatment; ^2^ SP = soluble protein; ^3^ NDFD = neutral detergent fiber digestibility, 30 h, % of NDF; ^4^ IVDMD = in vitro dry matter digestibility.

**Table 6:** Lactational performance of lactating dairy cows fed a total mixed ration (TMR) based on forages grown via control (CON) or soil and crop additive (SCA) agronomy program.

	TMR			
Measurement	CON	SCA	SEM	*p*< ^1^
Milk, kg/d	32.8	37.6	1.70	0.05
FCM, kg/d ^2^	33.7	39.0	2.58	0.14
ECM, kg/d ^3^	33.1	38.8	1.55	0.10
FPCM, kg/d ^4^	30.3	33.4	0.92	0.02
Fat, %	3.61	3.41	0.13	0.28
Protein, %	2.99	2.87	0.07	0.20
Lactose, %	4.93	4.97	0.09	0.70
SNF, % ^5^	8.80	8.75	0.13	0.74
Total solids, %	11.50	11.24	0.20	0.39
Fat, kg/d	1.18	1.27	0.06	0.27
Protein, kg/d	0.97	1.07	0.04	0.10
Lactose, kg/d	1.62	1.87	0.10	0.07
SNF, kg/d	2.89	3.33	0.14	0.03
Total solids, kg/d	3.78	4.21	0.19	0.10
SCS ^6^	3.99	3.70	0.42	0.64
MUN, mg/dL ^7^	14.4	15.0	0.42	0.32
DMI, kg/d ^8^	23.6	23.3	0.94	0.81
Initial BW, kg ^9^	631.4	640.4	19.9	0.40
Final BW, kg ^9^	646.7	649.5	19.7	0.92
Mean BW, kg ^9^	632.7	646.5	21.0	0.62
ADG, kg ^10^	0.15	0.11	0.12	0.80
DMI/BW, %	3.75	3.63	0.18	0.58
BCS ^11^	3.01	3.00	0.06	0.86
Milk/DMI, kg/kg	1.40	1.64	0.08	0.02
FCM/DMI, kg/kg	1.42	1.67	0.10	0.06
ECM/DMI, kg/kg	1.41	1.59	0.07	0.04

^1^ Probability of F-test for treatment; ^2^ FCM = fat-corrected milk = (0.4 × kg of milk) + (15 × kg of milk fat); ^3^ ECM = energy-corrected milk = (0.327 × kg, milk) + (12.95 × kg, milk fat) + (7.2 × kg, milk protein); ^4^ fat- and protein-corrected milk (FPCM): FPCM = milk, kg/d × ((0.1226 × fat, %) +(0.0776 × protein, %) + 0.2534); ^5^ SNF = solids-not-fat; ^6^ SCS = somatic cell score; ^7^ MUN = milk urea nitrogen; ^8^ DMI = dry matter intake; ^9^ BW = body weight; ^10^ ADG = average daily gain; ^11^ BCS = body condition score.

**Table 7:** Blood serum glucose, ruminal pH, ammonia–nitrogen, and volatile fatty acid (VFA) concentrations in lactating dairy cows fed a total mixed ration (TMR) based on forages grown via control (CON) or soil and crop additive (SCA) agronomy program.

	TMR			
Measurement	CON	SCA	SEM	*p*< ^1^
Glucose, mg/dL	60.0	60.6	1.59	0.76
pH	6.80	6.72	0.06	0.22
NH_3_-N, mg/dL	18.4	19.1	1.57	0.63
Total VFAs	97.4	98.9	2.15	0.57
Acetate, molar %	60.2	59.9	0.76	0.77
Propionate, molar %	21.5	22.8	0.67	0.10
Isobutyrate, molar %	1.47	1.44	0.03	0.17
Butyrate, molar %	13.0	12.0	0.25	0.01
Isovalerate, molar %	2.00	1.86	0.06	0.11
Valerate, molar %	1.74	1.84	0.07	0.24
Acetate/propionate	2.84	2.69	0.13	0.24
Acetate + butyrate/propionate	3.45	3.22	0.13	0.13

^1^ Probability of F-test for treatment.

**Table 8:** Total-tract nutrient digestibility in lactating dairy cows fed a total mixed ration (TMR) based on forages grown via control (CON) or soil and crop additive (SCA) agronomy crop production program.

	TMR			
Digestibility	CON	SCA	SEM	*p*< ^1^
DM, %	75.5	75.3	0.59	0.69
CP, %	74.0	75.8	1.42	0.20
NDF, %	48.5	54.7	2.78	0.03
ADF, %	48.3	54.4	2.54	0.02
Starch, %	97.9	98.6	0.27	0.06
Blood glucose, mg/dL	60.0	60.6	1.59	0.76

^1^ Probability of F-test for treatment.

## Data Availability

The data will be made available upon request from the corresponding author.

## Abbreviations

ADF = acid detergent fiber; ADG = average daily gain; ADIN = acid detergent-insoluble nitrogen; AIA = acid-insoluble ash; AOAC = Association of Official Analytical Chemists International; BCS = body condition score; BW = body weight; CON = control; CP = crude protein; DIM = days in milk; DM = dry matter; DMI = dry matter intake; DRTF = Dairy Research and Teaching Facility; ECM = energy-corrected milk; FCM = 4% fat-corrected milk; FPCM = fat- and protein-corrected milk; HC = hemicellulose; MUN = milk urea nitrogen; NE_L_ = net energy of lactation; NDF = neutral detergent fiber; NDIP = neutral detergent-insoluble nitrogen; NDFD = neutral detergent fiber digestibility; NH_3_-N = ammonia–nitrogen; NFC = non-fiber carbohydrate; NRC = National Research Council; RCBD = randomized complete block design; RFQ = relative forage quality; SCAs = soil and crop additives; SCCs = somatic cell counts; SCSs = linear somatic cell counts; SNF = solids-not-fat; SDSU = South Dakota State University; TMR = total mixed ration; VFAs = volatile fatty acids.

## References

[1] Van Soest P.J. (1982). Nutritional Ecology of the Ruminant.

[2] Casper D.P., Beede D.K. (2017). Carbohydrate nutrition. Large Dairy Herd Management.

[3] Casper D.P. How are we going to feed cows in the future?. Proceedings of the 7th Annual I-129 Dairy Conference.

[4] Vastolo A., Serrapica F., Cavallini D., Fusaro I., Atzori A.S., Todaro M. (2024). Editorial: Alternative and novel livestock feed; reducing environmental impact. Front. Vet. Sci..

[5] Gadeken D.L., Casper D.P. (2017). Evaluation of a high forage total mixed ration on the lactational performance of late lactation dairy cows. Transl. Anim. Sci..

[6] Acharya I.P., Casper D.P. (2020). Lactational response of early-lactation Holstein cows fed starch or floury corn silage. J. Dairy Sci..

[7] Casper D.P., Mertens D.R. (2007). Feed efficiency of lactating dairy cows is related to dietary energy density. J. Dairy Sci..

[8] Cavallini D., Mammi L.M.E., Biagi G., Fusaro I., Giammarco M., Formigoni A., Palmonari A. (2021). Effects of 00-rapeseed meal inclusion in Parmigiano Reggiano hay-based ration dairy cows’ production, reticular pH and fibre digestibility. Ital. J. Anim. Sci..

[9] Casper D.P. The application of feed on the dairy farm. Proceedings of the Southeast Dairy Herd Management Conference.

[10] Llamas-lamas G., Combs D.K. (1991). Effect of forage to concentrate ratio and intake level on utilization of early vegetative alfalfa silage by dairy cows. J. Dairy Sci..

[11] Martinez C.M., Chung Y.H., Ishler V.A., Bailey K.W., Varga G.A. (2009). Effects of dietary forage level and monensin on lactational performance, digestibility, and fecal excretion of nutrients and efficiency of feed nutrient utilization of Holstein dairy cows. J. Dairy Sci..

[12] Beauchemin K.A., Yang W.Z., Rode L.M. (2003). Effects of particle size of alfalfa-based dairy cow diets on chewing activity, ruminal fermentation, and milk production. J. Dairy Sci..

[13] Chase L.E., Grant R.J. High forage rations—What do we know?. Proceedings of the Cornell Nutrition Conference.

[14] Cherney D.J.R., Cherney J.H., Chase L.E. (2004). Lactation performance of Holstein cows fed fescue, orchardgrass, or alfalfa silage. J. Dairy Sci..

[15] Buxton D.R. (1996). Quality-related characteristics of forages as influenced by plant environmental and agronomic factors. Anim. Feed Sci. Technol..

[16] Brito A.F., Tremblay G.F., Bertrand A., Castongua Y., Belanger G., Lafreniere C., Martineau R., Berthiaume R. (2021). Omasal flow of nonstructural carbohydrates and nitrogenous compounds in lactating dairy cows fed diets containing timothy cut in the afternoon or morning. J. Dairy Sci..

[17] Bertrand A., Tremblay G.F., Pelletier S., Castonguay Y., Bélanger G. (2008). Yield and nutritive value of timothy as affected by temperature, photoperiod and time of harvest. Grass Forage Sci..

[18] Pelletier S., Tremblay G.F., Bélanger G., Bertrand A., Castonguay Y., Pageau D., Drapeau R. (2010). Forage nonstructural carbohydrates and nutritive value as affected by time of cutting and species. Agron. J..

[19] Pelletier S., Tremblay G.F., Lafrenière C., Bertrand A., Bélanger G., Castonguay Y., Rowsell J. (2009). Nonstructural carbohydrate concentrations in timothy as affected by N fertilization, stage of development, and time of cutting. Agron. J..

[20] Brito A.F., Tremblay G.F., Bertrand A., Castonguay Y., Bélanger G., Michaud G., Lafrenière C., Martineau R., Berthiaume R. (2016). Performance and nitrogen use efficiency in mid-lactation dairy cows fed timothy cut in the afternoon or morning. J. Dairy Sci..

[21] Brito A.F., Tremblay G.F., Lapierre H., Bertrand A., Castonguay Y., Bélanger G., Michaud R., Benchaar C., Ouellet D.R., Berthiaume R. (2009). Alfalfa cut at sundown and harvested as baleage increases bacterial protein synthesis in late-lactation dairy cows. J. Dairy Sci..

[22] Brito A.F., Tremblay G.F., Bertrand A., Castonguay Y., Bélanger G., Michaud R., Lapierre H., Benchaar C., Petit H.V., Ouellet D.R. (2008). Alfalfa cut at sundown and harvested as baleage improves milk yield of late-lactation dairy cows. J. Dairy Sci..

[23] National Research Council (2001). Nutrient Requirements of Dairy Cattle.

[24] Chen S.-K., Subler S., Edwards C.A. (2002). Effects of agricultural biostimulants on soil microbial activity and nitrogen dynamics. Appl. Soil Ecol..

[25] Chen S.-K., Subler S., Edwards C.A. (2003). The influence of two agricultural biostimulants on nitrogen transformations, microbial activity, and plant growth in soil microcosms. Soil Biol. Biochem..

[26] Tian S., Lu L., Xie R., Zhang M., Jemstedt J.A., Hou D., Ramsier C., Brown P.H. (2015). Supplemental macronutrients and microbial fermentation products improve the uptake and transport of foliar applied zinc in sunflower (*Helianthus annuus* L.) plant: Studies using micro X-ray florescence. Front. Plant Sci..

[27] ADSA, ASAS, PSA (2020). Guide for the Care and Use of Agricultural Animals in Research and Teaching.

[28] AOAC International (2019). Official Methods of Analysis.

[29] Wildman E.F., Jones G.M., Wagner P.E., Boman R.L., Troutt H.F., Lesch T.N. (1982). A dairy cow body condition scoring system and its relationship to standard production characteristics. J. Dairy Sci..

[30] Edmonson A.J., Lean I.J., Weaver L.D., Farver T., Webster G. (1989). A body condition scoring chart for Holstein dairy cows. J. Dairy Sci..

[31] Krishnamoorthy U., Muscato T.V., Sniffen C.J., Van Soest P.J. (1982). Nitrogen fractions in selected feedstuffs. J. Dairy Sci..

[32] Hall M.B. (2009). Determination of starch, including maltooligosaccharides, in animal feeds: A comparison of methods and a recommend method for AOAC collaborative study. J. AOAC Int..

[33] Damon C. (1966). Hawk’s Physiological Chemistry.

[34] Marten G.C., Barnes R.F. (1980). Prediction of energy digestibility of forages with in vitro rumen fermentation and fungal enzyme systems. Standardization of Analytical Methodology for Feeds: Proceedings of a Worship held in Ottawa, Canada, 12–14 March 1979.

[35] Van Soest P.J., Robertson J.B., Lewis B.A. (1991). Methods for dietary fiber, neutral detergent fiber, and nonstarch polysaccharides in relation to animal nutrition. J. Dairy Sci..

[36] El Rassi Z. (1996). High Performance Capillary Electrophoresis of Carbohydrates.

[37] Cancalon P.F. (1993). Rapid monitoring of fruit juice adulteration by capillary electrophoresis. Liq. Chromatogr. Gas Chromatogr..

[38] Rohweder D.A., Barnes R.F., Jorgensen N. (1978). Proposed hay grading standards based on laboratory analyses for evaluating quality. J. Anim. Sci..

[39] DuBois M., Gilles K.A., Hamilton J.K., Rebers P.A., Smith F. (1956). Colorimetric method for determination of sugars and related substances. Anal. Chem..

[40] Berthelot A. (1979). Spectrophotometric analysis of urea. Clin. Chem..

[41] Spaniol J.S., Oltramari C.E., Locatelli M., Volpato A., Campigotto G., Stefani L.M., Da Silva A.S. (2015). Influence of probiotic on somatic cell count in milk and immune system of dairy cows. Comp. Clin. Pathol..

[42] Orth R. (1992). Sample Day and Lactation Report: DHIA 200 Fact Sheet A-2.

[43] International Dairy Federation (2015). A Common Carbon Footprint Approach for the Dairy Sector—The IDF Guide to Standard Life Cycle Assessment Methodology.

[44] Chaney A.L., Marbach E.P. (1962). Modified reagents for determination of urea and ammonia. Clin. Chem..

[45] Erwin E.S., Marco G.J., Emery E.M. (1961). Volatile fatty acid analysis of blood and rumen fluid by gas chromatography. J. Dairy Sci..

[46] Van Keulen J., Young B.A. (1977). Evaluation of acid-insoluble ash as a natural market in ruminant digestibility studies. J. Anim. Sci..

[47] Steel R.G.D., Torrie J.H. (1980). Principles and Procedures of Statistics.

[48] Putman D., Orloff S., Ackerly T. Agronomic practices and forage quality. Proceedings of the 2000 National Alfalfa Symposium.

[49] Al-Khatib K., Parker K., Fuerst E.P. (1992). Alfalfa (*Medicago sativa*) response to simulated herbicide spray drift. Weed Technol..

[50] Lissbrant S., Stratton S., Cunningham S.M., Brouder S.M., Volenec J.J. (2009). Impact of long-term phosphorus and potassium fertilization on alfalfa nutritive value-yield relationships. Crop Sci..

[51] Casper D.P., Srivastava S., Strayer B. (2017). Feeding a calf starter containing highly digestible corn may improve calf growth. Trans. Anim. Sci..

[52] Dado R.G., Allen M.S. (1996). Enhanced intake and production of cows offered ensiled alfalfa with higher neutral detergent fiber digestibility. J. Dairy Sci..

[53] Aguerre M.J., Wattiaux M.A., Powell J.M., Broderick G.A., Arndt C. (2001). Effect of forage-to-concentrate ratio in dairy cows diets on emission of methane, carbon dioxide, and ammonia, lactation performance, and manure excretion. J. Dairy..

[54] Ruiz T.M., Bernal E., Staples C.R., Sollenberger L.E., Gallaher R.N. (1995). Effects of dietary neutral detergent fiber concentration and forage source on performance of lactating cows. J. Dairy Sci..

[55] Arendt C., Powell J.M., Aguerre M.J., Watiaux M.A. (2015). Performance, digestion, nitrogen balance, and emission of manure ammonia, enteric methane, and carbon dioxide in lactating cows fed diets with varying alfalfa silage-to-corn silage ratios. J. Dairy Sci..

[56] Cant J.P., Trout D.R., Quiao F., Purdie N.G. (2002). Milk synthetic response of the bovine mammary gland to an increase in the local concentration of arterial glucose. J. Dairy Sci..

[57] Akers R.M. (2016). Lactation and the Mammary Gland.

[58] Neville M.C., Allen J.C., Watters C., Neville M.C., Neifert M.R. (1983). The Mechanisms of Milk Secretion. Lactation.

[59] Raffrenato E., Fievisohn R., Cotanch K.W., Grant R.J., Chase L.E., Van Amburgh M.E. (2017). Effect of lignin linkages with other plant cell wall components on in vitro and *in vivo* neutral detergent fiber digestibility and rate of digestion of grass forages. J. Dairy Sci..

[60] Seymour W.M., Campbell D.R., Johnson Z.B. (2005). Relationships between rumen volatile fatty acid concentrations and milk production in dairy cows: A literature study. Anim. Feed Sci. Technol..

